# Relationship between tobacco use, alcohol consumption and non-communicable diseases among women in India: evidence from National Family Health Survey-2015-16

**DOI:** 10.1186/s12889-022-13191-z

**Published:** 2022-04-11

**Authors:** Vivek K. Mishra, Shobhit Srivastava, T. Muhammad, P. V. Murthy

**Affiliations:** 1 Department of Population Studies, Sri Venkateswara University, Tirupati, Andhra Pradesh 517 502 India; 2 International Institute for Population Sciences, Mumbai, Maharashtra 400088 India; 3 Department of Population Studies and Social Work, College of Arts, Sri Venkateswara University, Tirupati, Andhra Pradesh 517502 India

**Keywords:** Non-communicable disease, Tobacco, Alcohol, Population attributable risk, Women, India

## Abstract

**Background:**

Based on an increased prevalence of diabetes, asthma and hypertension among women in reproductive age, understanding the risk factors of non-communicable diseases (NCDs) is crucial to inform policy and program interventions to address the problem. In this study, we empirically assessed the associations of behavioural factors such as alcohol consumption and tobacco use and a variety of socioeconomic characteristics with prevalence of NCDs in adult women.

**Methods:**

The data were derived from the National Family Health Survey conducted in 2015–16. The effective sample size for the present paper was 699,686 women aged 15–49 years in India. Descriptive statistics along with bivariate analysis were conducted to find the preliminary results. Additionally, multivariable logistic regression analysis was conducted to find the relationship between NCDs and behavioural factors such as alcohol consumption and tobacco use. Moreover, population attributable risk was estimated in the present study.

**Results:**

It was revealed that 15.9% of women had any of the NCDs. A proportion of 0.8% of women smoked tobacco whereas 5.5% of women consumed smokeless tobacco. Also, a proportion of 1.2% of women consumed alcohol in the current study. The odds of having NCDs among women who smoked tobacco, consumed smokeless tobacco and consume alcohol were 16, 8 and 20% significantly higher than the odds of having NCDs among women who did not smoke tobacco, consume smokeless tobacco and consume alcohol respectively. The population attributable risk of having NCDs was 1.8% (*p* < 0.001) for women who smoked, 0.8% (*p* < 0.001) for women who consumed smokeless tobacco and 2.2% (*p* < 0.001) for women who consumed alcohol. Besides, the odds of having NCDs among overweight and obese women were 2.25 and 3.60 times greater than the odds of having NCDs among women who were underweight.

**Conclusion:**

The findings revealed that smoking and using smokeless tobacco and alcohol consumption were risk factors of NCDs in women. The findings also alarm the focus of maternal and child health programs on NCDs’ risk factors like maternal obesity, due to their adverse health consequences on their children too. Also, the coexistence of higher levels of tobacco use and alcohol consumption requires different strategies to address the vulnerability of women towards NCDs, including screening and early detection of NCDs especially among those who smoke or chew tobacco and consume alcohol.

**Supplementary Information:**

The online version contains supplementary material available at 10.1186/s12889-022-13191-z.

## Background

More than two-thirds of the deaths worldwide are caused by non-communicable diseases (NCDs), whereas, three-fourth of such mortality occurs in less-developed countries [1]. Smoked and smokeless tobacco which are highly prevalent in South Asia along with problematic alcohol consumption is responsible for a large number of diseases and deaths [2].

A growing body of literature suggests that women are more likely to experience the co-occurrence of behavioural risk factors thus increasing the risk of NCDs among them and in the future generation [3–5]. Multiple studies in different socio-cultural settings show that higher consumption of alcohol increase the risk of coronary artery disease and related mortality [6–9]. Further, the socioeconomic determinants of NCDs among women are well documented with a higher risk among women in poor resource settings [10–13]. The low-income women were more likely to smoke and had a higher prevalence of many chronic diseases and related risk factors than higher-income mothers [14, 15]. Also, the prevalence of overweight and central obesity which are risk factors for NCDs have been found to be consistently higher in women in India than men in multiple studies [16–18].

However, studies focusing on behavioural risk factors of NCDs with a wider sample of Indian women are still lacking. Based on an increased prevalence of diabetes, asthma and hypertension among women in reproductive age, understanding the risk factors of NCDs is crucial to inform policy and program interventions to address the problem. In this study, we empirically assess the associations of behavioural factors such as alcohol consumption and tobacco use and a variety of socioeconomic characteristics with the prevalence of NCDs. In addition, we estimate the population-attributable risk (PAR) of NCDs due to tobacco, alcohol and other exposures among women in India.

## Methods

### Data

The data were derived from the National Family Health Survey (NFHS-4), the fourth in the NFHS series conducted in 2015–16 [19]. It provides information on population, health, and nutrition of people in India and each state and union territory of the country. All four rounds of NFHS survey have been conducted under the stewardship of the Ministry of Health and Family Welfare (MoHFW), Government of India. MoHFW designated the International Institute for Population Sciences (IIPS), Mumbai, as the nodal agency for conducting the surveys. Decisions about the overall sample size required for NFHS-4 were guided by several considerations, paramount among which was the need to produce indicators at the district, state/union territory and national levels, as well as separate estimates for urban and rural areas in 157 districts that have 30–70% of the population living in urban areas as per the 2011 census, with a reasonable level of precision. The NFHS-4 sample is a stratified two-stage sample [19]. The 2011 census served as the sampling frame for the selection of Primary Sampling Units (PSUs). PSUs were villages in rural areas and Census Enumeration Blocks (CEBs) in urban areas. PSUs with fewer than 40 households were linked to the nearest PSU. Within each rural stratum, villages were selected from the sampling frame with probability proportional to size [19]. In each stratum, six approximately equal substrata were created by crossing three substrata, each created based on the estimated number of households in each village, with two substrata, each created based on the percentage of the population belonging to Scheduled Castes and Scheduled Tribes. Four survey questionnaires (Household Questionnaire, Woman’s Questionnaire, Man’s Questionnaire, and Biomarker Questionnaire) were canvassed in 17 local languages using Computer Assisted Personal Interviewing (CAPI). In the interviewed households, 723,875 eligible women aged 15–49 years were identified for individual women’s interviews [19]. Interviews were completed with 699,686 women, with a response rate of 97%. In all, there were 122,051 eligible men aged 15–54 years in households selected for the state module. Interviews were completed with 112,122 men, with a response rate of 92% [19]. The effective sample size for the present study was 699,686 women aged 15–49 years in India.

### Variable description

#### Outcome variable

The outcome variable was ‘presence of NCDs’ which was recoded as no and yes. The diseases considered for measuring NCDs were hypertension, diabetes, asthma, heart disease and cancer. Blood pressure was measured among women aged 15–49 using an Omron Blood Pressure Monitor to determine the prevalence of hypertension [19]. Blood pressure measurements for each respondent were taken three times with an interval of 5 min between readings [19]. Hypertension is defined as when an individual had average systolic blood pressure of more than or equals to 140 mmHg and/or diastolic blood pressure of more than or equals to 90 mmHg [20]. If the random blood glucose level exceeds 140 mg/dl, the person is termed diabetic. The FreeStyle Optium H glucometer with glucose test strips was used to measure random blood glucose for women aged 15–49 using a finger-stick blood sample [19]. Further, asthma, heart disease and cancer were self-reported [21]. If the respondent had any of the above diseases, they were considered to be having NCDs.

#### Explanatory variable

The explanatory variables were selected on the basis of extensive literature review. The variables were divided into three sections that are behavioural, individual and household characteristics.

### Behavioural characteristics

i. Cigarettes, *bidis*, *cigars, hookah*, *gutkha/paan masala*, *paan* and *khaini* are tobacco products commonly consumed in India. The variable ‘smoke tobacco’ was generated using the questions a. Do you currently smoke cigarettes? b. Do you currently smoke *bidis*? C. Do you currently smoke *cigar*? and e. Do you currently smoke *hookah*? All the responses were recoded as no and yes. And if the female respondents smoked any of these products, they were coded as yes and otherwise no.
ii. The variable ‘consume smokeless tobacco’ was generated using the questions a. Do you currently chew tobacco? b. Do you currently consume *gutkha/paan masala* with tobacco? c. Do you currently consume *paan* with tobacco? and e. Do you currently consume *khaini*? All the responses were recoded as no and yes. And if the female respondents consumed any of these products, they were coded as yes and otherwise no.
iii. Women who consume alcohol were coded as no and yes. The variable was generated using the question “Do you currently drink alcohol?” the response was coded as no and yes.

### Individual characteristics

Age was grouped into 15–24 years, 25–34 years and 35–49 years. Educational status was categorized as not educated, primary, secondary and higher. Working status was coded as no and yes. The variable on working status was asked under state module hence cannot be used for multi-variate analysis. Marital status was coded as never married, currently married and others. Others included those who were divorced, separated or widowed. Media exposure was coded as not exposed and exposed. The variable was generated using the question on whether women watched television, read newspaper or listened to radio. If the response was affirmative to any of these, it was coded as yes otherwise no. Body mass index (BMI) was recoded as underweight (less than 18.5), normal (18.5 to 24.9), overweight (25–29.9) and obese (30 and above) [22].

### Household characteristics

The variable wealth status was generated using the information given in the NFHS 2015–16 survey. Households were given scores based on the number and kinds of consumer goods they own, ranging from a television to a car or bicycle, and housing characteristics such as toilet facilities, source of drinking water, and flooring materials. These scores are derived using principal component analysis (PCA). National wealth quintiles are compiled by assigning the household score to each usual (de jure) household member, ranking each person in the household population by their score, and then dividing the distribution into five equal categories, each with 20% of the population [23]. The wealth status was coded as poorest, poorer, middle, richer and richest.

Religion was coded as Hindu, Muslim, Christian and others. Others included Buddhist, Sikh, Jain, etc. Caste was coded as Scheduled Tribe, Scheduled Caste, Other Backward Class and others [23]. Others include those who were identified as having higher social status [24, 25]. Place of residence was coded as urban and rural. Regions of India were coded as North, Central, East, North-East, West and South [19].

### Statistical analysis

All the analyses have been conducted using STATA 14. Descriptive statistics along with bivariate analysis were performed at the initial stage. Chi-square test was used to find the significance level for the prevalence estimates of NCDs by background variables. Additionally, multivariable logistic regression analysis [26] was used to estimate the extent of association between NCDs and behavioural factors along with other individual and household factors.

The binary logistic regression model is usually put into a more compact form as follows:

$$\mathrm{Logit}\ \left[\mathrm{P}\left(\mathrm{Y}=1\right)\right]={\beta}_0+\beta \ast X$$

The parameter *β*_0_ estimates the log odds of NCDs for the reference group, while *β* estimates the maximum likelihood, the differential log odds of NCDs associated with a set of predictors X, as compared to the reference group. Variance inflation factor (VIF) was estimated to check the multicollinearity among the variables used in the study [27]. The *svyset* command in STATA 14 was used to control the analysis for complex survey design. Additionally, this command also incorporated the weights which make the estimates representative.

Model-2, model-3 and model-4 reveal the combined effects of smoking and consuming smokeless tobacco, smoking tobacco and alcohol consumption and consuming smokeless tobacco and alcohol consumption. An “interaction variable” is a variable constructed from an original set of variables to represent either all of the interaction present or some part of it. In exploratory statistical analyses, it is common to use products of original variables as the basis of testing whether interaction is present with the possibility of substituting other more realistic interaction variables at a later stage. When there are more than two explanatory variables, several interaction variables are constructed, with pairwise-products representing pairwise-interactions and higher order products representing higher order interactions [28–30].

Thus, for a response *Y* and two variables *x*_1_ and *x*_2,_ an *additive* model would be:

$$\mathrm{Y}=\alpha +{\beta}_1{\mathrm{x}}_1+{\beta}_2{\mathrm{x}}_2+{\varepsilon}_0$$

In contrast to this,

$$\mathrm{Y}=\alpha +{\beta}_1{\mathrm{x}}_1+{\beta}_2{\mathrm{x}}_2+\left({\beta}_3{\mathrm{x}}_{\mathrm{s}}\ast {\mathrm{x}}_{\mathrm{a}}\right)\ {\varepsilon}_0$$

Where, Y is dependent variable (various NCDs) and α is intercept, x_1_ is individual level independent variable, x_2_ is individual level independent variable, x_a_ is alcohol users, x_s_ is smokers, (β_3_ x_s_ * x_a_) is the interaction of alcohol and smoking and ε_0_ is error. Often, models are presented without the interaction term d (x_1 *_ x_2_), but this confounds the main effect and interaction effect (i.e., without specifying the interaction term, it is possible that any main effect found is actually due to an interaction) [31].

Further, population attributable risk (PAR) was calculated to verify the extent of risk for NCDs among women who were exposed to negative behavioural factors i.e., smoking tobacco, consuming smokeless tobacco and alcohol [32]. The “regpar” command in STATA was used to calculate the PAR. The regpar generates confidence intervals for both population attributable risks and scenario proportions [33]. After an estimating command that interprets projected values as conditional proportions, such as logit, logistic, probit, or generalized linear model, regpar can be utilised [33]. It calculates two scenario proportions: a baseline (“Scenario 0”) and a fantasy (“Scenario 1”), in which one or more exposure variables are presumed to be set to specific values (usually zero) and all other predictor variables in the model remain unchanged. It also calculates the difference between the proportions in Scenario 0 and Scenario 1. This difference is referred to as the population attributable risk (PAR), and it shows the risk associated with living in Scenario 0 rather than Scenario 1 [33].

## Results

Table 1 presents the socioeconomic profile of women aged 15–49 years in India. A proportion of 0.8% of the women smoked tobacco whereas 5.5% of women consumed smokeless tobacco. Also, a proportion of 1.2% of women consumed alcohol in the current study.

**Table 1:** Socio-economic profile of the women aged 15–49 years in India, NFHS 2015–16

Background characteristics	Sample	Percentage
**Behavioural characteristics**		
**Smoke tobacco**		
No	694,274	99.23
Yes	5412	0.77
**Chew tobacco**		
No	661,453	94.54
Yes	38,233	5.46
**Alcohol consumption**		
No	691,048	98.77
Yes	8638	1.23
**Individual characteristics**		
**Age (in years)**		
15–24	244,518	34.95
25–34	211,812	30.27
35–49	243,357	34.78
**Educational status**		
Not educated	192,135	27.46
Primary	87,233	12.47
Secondary	331,037	47.31
Higher	89,281	12.76
**Working status**^**b**^		
No	92,996	76.01
Yes	29,355	23.99
**Marital status**		
Never married	159,035	22.73
Currently married	511,373	73.09
Others	29,279	4.18
**Media exposure**		
Not exposed	132,158	18.89
Exposed	567,528	81.11
**Body Mass Index**^**a**^		
Underweight	153,331	21.91
Normal	390,201	55.77
Overweight	105,038	15.01
Obese	34,269	4.90
**Household characteristics**		
**Wealth status**		
Poorest	124,054	17.73
Poorer	136,900	19.57
Middle	143,814	20.55
Richer	147,978	21.15
Richest	146,939	21.00
**Religion**		
Hindu	563,739	80.57
Muslim	96,461	13.79
Christian	16,620	2.38
Others	22,866	3.27
**Caste**		
Scheduled Caste	142,619	20.38
Scheduled Tribe	64,144	9.17
Other Backward Class	303,837	43.42
Others	189,086	27.02
**Place of residence**		
Urban	242,225	34.62
Rural	457,461	65.38
**Regions**		
North	95,098	13.59
Central	165,474	23.65
East	154,698	22.11
North East	24,615	3.52
West	100,535	14.37
South	159,266	22.76
Total	699,686	100.00

^a^Sample is low due to missing cases

^b^The question was asked on state module therefore sample is low

**Fig. 1:**
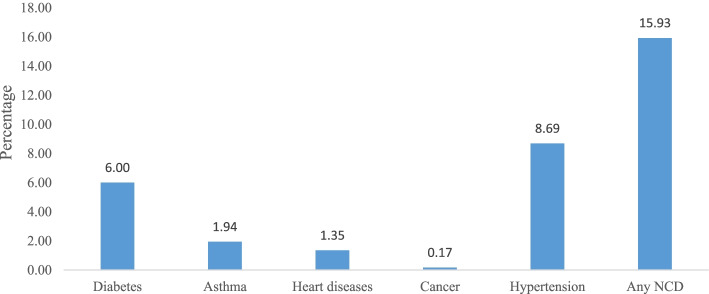
Percentage of NCDs among women aged 15–49 years in India, NFHS 2015–16

**Fig. 2:**
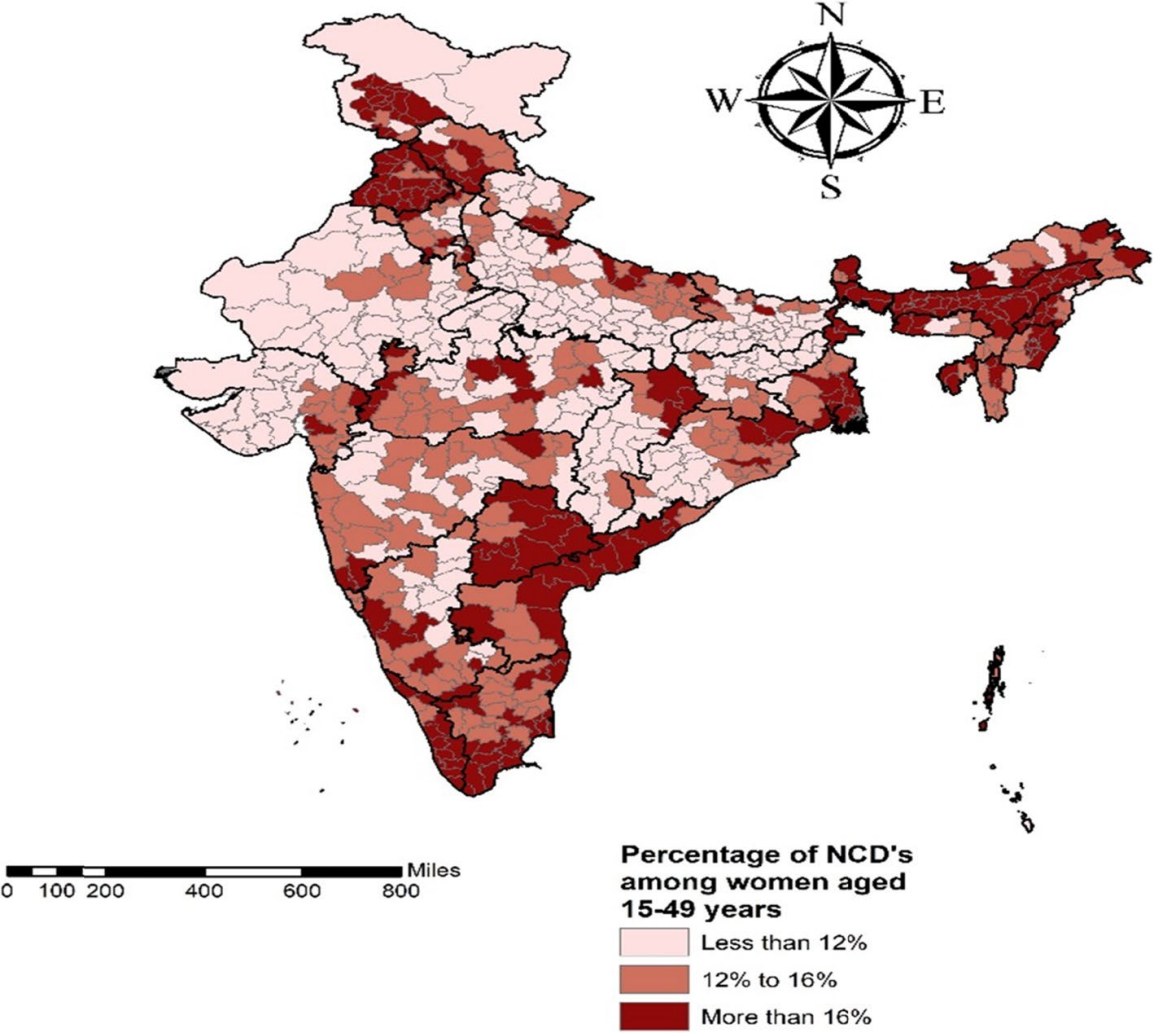
Prevalence of NCDs among women aged 15–49 years in states of India, NFHS 2015–16

Figure 1 presents percentage of NCDs among women aged 15–49 years. A proportion of 6.0% of women were diabetic and 8.7% were hypertensive. Additionally, 1.9% had asthma, 1.4% had heart diseases and 0.2% had cancer. Moreover, 15.9% of women had any of the NCDs. Further, Fig. 2 reveals that the prevalence of NCDs (more than 16%) was concentrated in the states of Jammu and Kashmir, Punjab, Himachal Pradesh, Chhattisgarh, North Eastern states and in almost all the Southern states of India.

Table 2 presents percentage of women aged 15–49 years having NCDs by their background characteristics. It was revealed that 25.1% of women who smoked vs 15.9% who did not smoke had NCDs and, 22.8% who consumed smokeless tobacco vs 15.5% who did not consume smokeless tobacco had NCDs. Also, a higher percentage of women who consumed alcohol (24.8%) had NCDs in comparison to those who did not consume alcohol (15.8%).

**Table 2:** Percentage of women aged 15–49 having NCDs by their background characteristics

Background characteristics	No NCD	Any NCD	***p***-value
**Behavioural characteristics**	%	%	
**Smoke tobacco**			0.001
No	84.14	15.86	
Yes	74.94	25.06	
**Chew tobacco**			0.001
No	84.46	15.54	
Yes	77.24	22.76	
**Alcohol consumption**			0.001
No	84.18	15.82	
Yes	75.23	24.77	
**Individual characteristics**			
**Age (in years)**			0.001
15–24	93.58	6.42	
25–34	86.92	13.08	
35–49	72.03	27.97	
**Educational status**			0.001
Not educated	79.94	20.06	
Primary	80.97	19.03	
Secondary	86.14	13.86	
Higher	88.3	11.7	
**Working status**			0.001
No	85.06	14.94	
Yes	82.21	17.79	
**Marital status**			0.001
Never married	93.18	6.82	
Currently married	81.8	18.2	
Others	74.21	25.79	
**Media exposure**			0.001
Not exposed	84.25	15.75	
Exposed	84.02	15.98	
**Body Mass Index**			0.001
Underweight	90.14	9.86	
Normal	86.17	13.83	
Overweight	72.64	27.36	
Obese	61.85	38.15	
**Household characteristics**			
**Wealth status**			0.001
Poorest	85.91	14.09	
Poorer	85.25	14.75	
Middle	84.51	15.49	
Richer	82.34	17.66	
Richest	82.71	17.29	
**Religion**			0.001
Hindu	84.4	15.6	
Muslim	83.18	16.82	
Christian	80.23	19.77	
Others	82.52	17.48	
**Caste**			0.001
Scheduled Caste	84.78	15.22	
Scheduled Tribe	84.28	15.72	
Other Backward Class	84.59	15.41	
Others	82.61	17.39	
**Place of residence**			0.001
Urban	82.97	17.03	
Rural	84.65	15.35	
**Regions**			0.001
North	85.4	14.6	
Central	85.76	14.24	
East	83.72	16.28	
North East	79.22	20.78	
West	84.85	15.15	
South	82.11	17.89	
**Total**	84.07	15.93	

Table 3 presents the logistic regression estimates for women having NCDs by their background characteristics. Women aged 35–49 years had significantly higher odds of having NCDs in comparison to women aged 15–24 years. The odds of having NCDs among women with higher educational status were 25% higher than the odds of having NCDs among women who were not educated. The odds of having NCDs among women who were divorced/separated/widowed were 16% higher than the odds of having NCDs among women who were never married. Similarly, women who smoked tobacco had 16% significantly higher odds of having NCDs than women who did not smoke tobacco. Women who consumed smokeless tobacco had 8% significantly higher odds of having NCDs than women who did not consume smokeless tobacco. Besides, the odds of having NCDs among women who consumed alcohol were 20% significantly higher than those who did not consume alcohol. Model-2, model-3 and model-4 reveal the combined effect of tobacco use and alcohol consumption on having NCDs among women in India. The odds of having NCDs among women who smoked and consumed smokeless tobacco were 8% significantly higher than the odds of having NCDs among women who did not consume any of these. Women who smoked tobacco and consumed alcohol had 42% significantly higher odds of having NCDs than women who did not consume any of these. On the other hand, the odds of having NCDs among women who consumed smokeless tobacco and consumed alcohol were 32% significantly higher than the odds of having NCDs among women who did not consume any of these. Also, women who were overweight and obese had 2.25- and 3.60-times greater odds of having NCDs than women who were underweight as revealed from model-1. Table S1 in supplementary file represented the regression estimates for individual diseases, i.e., diabetes, asthma, heart disease, cancer and hypertension.

**Table 3:** Multivariable logistic regression estimates for NCDs by background characteristics among women aged 15–49 years in India, 2015–16

Background characteristics	Model-1	Model-2	Model-3	Model-4
	AOR (95% CI)	AOR (95% CI)	AOR (95% CI)	AOR (95% CI)
**Behavioural characteristics**				
**Smoke tobacco**				
No	Ref.			Ref.
Yes	1.16*(1.1,1.22)			1.16*(1.1,1.22)
**Consume smokeless tobacco**				
No	Ref.		Ref.	
Yes	1.08*(1.05,1.1)		1.08*(1.05,1.1)	
**Alcohol consumption**				
No	Ref.	Ref.		
Yes	1.20*(1.16,1.25)	1.20*(1.16,1.25)		
**Individual characteristics**				
**Age (in years)**				
15–24	Ref.	Ref.	Ref.	Ref.
25–34	1.79*(1.75,1.84)	1.79*(1.75,1.84)	1.79*(1.75,1.84)	1.79*(1.75,1.84)
35–49	4.11*(4.01,4.21)	4.10*(4,4.21)	4.10*(4,4.21)	4.10*(4,4.21)
**Educational status**				
Not educated	Ref.	Ref.	Ref.	Ref.
Primary	1.02*(1,1.04)	1.02*(1,1.05)	1.02*(1,1.05)	1.02*(1,1.05)
Secondary	0.89*(0.88,0.91)	0.90*(0.88,0.91)	0.90*(0.88,0.91)	0.90*(0.88,0.91)
Higher	0.75*(0.73,0.77)	0.75*(0.73,0.77)	0.75*(0.73,0.77)	0.75*(0.73,0.77)
**Marital status**				
Never married	Ref.	Ref.	Ref.	Ref.
Currently married	0.99(0.96,1.01)	0.99(0.96,1.01)	0.99(0.96,1.01)	0.99(0.96,1.01)
Others	1.16*(1.11,1.20)	1.16*(1.11,1.20)	1.16*(1.11,1.20)	1.16*(1.11,1.20)
**Media exposure**				
Not exposed	Ref.	Ref.	Ref.	Ref.
Exposed	1.04*(1.02,1.07)	1.04*(1.02,1.07)	1.04*(1.02,1.07)	1.04*(1.02,1.07)
**Body Mass Index**				
Underweight	Ref.	Ref.	Ref.	Ref.
Normal	1.18*(1.15,1.2)	1.18*(1.15,1.2)	1.18*(1.15,1.2)	1.18*(1.15,1.2)
Overweight	2.25*(2.2,2.31)	2.25*(2.2,2.31)	2.25*(2.2,2.31)	2.25*(2.2,2.31)
Obese	3.60*(3.49,3.72)	3.60*(3.49,3.72)	3.60*(3.49,3.72)	3.60*(3.49,3.72)
**Household characteristic’s**				
**Wealth status**				
Poorest	Ref.	Ref.	Ref.	Ref.
Poorer	1.04*(1.01,1.06)	1.04*(1.01,1.06)	1.04*(1.01,1.06)	1.04*(1.01,1.06)
Middle	1.05*(1.03,1.08)	1.05*(1.03,1.08)	1.05*(1.03,1.08)	1.05*(1.03,1.08)
Richer	1.12*(1.09,1.16)	1.13*(1.09,1.16)	1.13*(1.09,1.16)	1.13*(1.09,1.16)
Richest	1.10*(1.07,1.14)	1.10*(1.07,1.14)	1.10*(1.07,1.14)	1.10*(1.07,1.14)
**Religion**				
Hindu	Ref.	Ref.	Ref.	Ref.
Muslim	1.15*(1.12,1.17)	1.15*(1.12,1.17)	1.15*(1.12,1.17)	1.15*(1.12,1.17)
Christian	0.98(0.95,1.01)	0.98(0.95,1.01)	0.98(0.95,1.01)	0.98(0.95,1.01)
Others	1.03(1,1.06)	1.03(1,1.06)	1.03(1,1.06)	1.03(1,1.06)
**Caste**				
Scheduled Caste	Ref.	Ref.	Ref.	Ref.
Scheduled Tribe	1.03*(1,1.06)	1.03*(1,1.06)	1.03*(1,1.06)	1.03*(1,1.06)
Other Backward Class	0.95*(0.93,0.97)	0.95*(0.93,0.97)	0.95*(0.93,0.97)	0.95*(0.93,0.97)
Others	1.04*(1.02,1.07)	1.04*(1.02,1.07)	1.04*(1.02,1.07)	1.04*(1.02,1.07)
**Place of residence**				
Urban	Ref.	Ref.	Ref.	Ref.
Rural	1.04*(1.02,1.06)	1.04*(1.02,1.06)	1.04*(1.02,1.06)	1.04*(1.02,1.06)
**Regions**				
North	Ref.	Ref.	Ref.	Ref.
Central	1.06*(1.04,1.09)	1.06*(1.04,1.09)	1.06*(1.04,1.09)	1.06*(1.04,1.09)
East	1.13*(1.11,1.16)	1.13*(1.11,1.16)	1.13*(1.11,1.16)	1.13*(1.11,1.16)
North East	1.48*(1.44,1.52)	1.48*(1.44,1.52)	1.48*(1.44,1.52)	1.48*(1.44,1.52)
West	1(0.98,1.03)	1.01(0.98,1.04)	1.01(0.98,1.04)	1.01(0.98,1.04)
South	1.09*(1.06,1.12)	1.09*(1.06,1.12)	1.09*(1.06,1.12)	1.09*(1.06,1.12)
**Smoke tobacco # Consume smokeless tobacco**				
No # no		Ref.		
Yes # no		1.09*(1.06,1.11)		
No # yes		1.23*(1.16,1.31)		
Yes # yes		1.08*(1.03,1.19)		
**Smoke tobacco # alcohol consumption**				
No # no			Ref.	
Yes # no			1.2*(1.15,1.25)	
No # yes			1.15*(1.09,1.22)	
Yes # yes			1.42*(1.23,1.62)	
**Consume smokeless tobacco # alcohol consumption**				
No # no				Ref.
Yes # no				1.18*(1.13,1.25)
No # yes				1.07*(1.05,1.1)
Yes # yes				1.32*(1.25,1.41)

*Ref* Reference, *AOR* Adjusted odds ratio, *CI* Confidence interval, *if *p* < 0.05

^#^Interaction term

Table 4 presents the PAR of presence of NCDs among women who smoked tobacco, consumed smokeless tobacco and consumed alcohol. The proportion of NCDs that was attributable to smoked tobacco was 17.8% in comparison to 15.9% who did not smoke tobacco. The difference between two situations is PAR, which was measured to be 1.8% (*p* < 0.001). Similarly, the PAR for women who consumed smokeless tobacco was 0.8% (*p* < 0.001) and it was 2.2% (*p* < 0.001) among women who consumed alcohol. Tables S2, S3, S4, S5, S6 and S7 display the results for PAR for diabetes, asthma, heart disease, cancer and hypertension separately.

**Table 4:** Population attributable risk for NCDs among women aged 15–49 years

Population attributable risk (PAR)	
Behavioural factors	NCDs
**Smoke tobacco**	
No	0.159*(0.159, 0.160)
Yes	0.178*(0.171, 0.185)
PAR	0.018*(0.011, 0.027)
**Consume smokeless tobacco**	
No	0.159*(0.159, 0.169)
Yes	0.168*(0.165, 0.170)
PAR	0.008*(0.005, 0.010)
**Alcohol consumption**	
No	0.159*(0.159, 0.160)
Yes	0.182*(0.177, 0.188)
PAR	0.022*(0.017, 0.028)

The analysis was controlled for individual and household characteristics

*CI* Confidence Interval,  *if *p* < 0.05

## Discussion

In this study using nationally representative secondary data in India, we extensively explored the prevalence of major risk factors of NCDs which include tobacco use, alcohol consumption, overweight, and obesity among women of reproductive age. Furthermore, we investigated the population attributable risk of behavioural factors on the prevalence of NCDs to understand the pattern of the problem and how best to prevent and control it. The findings of this study revealed that a large number of women in India were having any of the NCDs. The findings in our study showed a higher prevalence of hypertension, diabetes, asthma and heart disease among women of reproductive age in India compared to other earlier surveys, with 14% of them having any of the NCDs. These findings were comparable to the reports from previous studies in India [34–36].

The present study also revealed that smoking and consuming of tobacco products and alcohol consumption were associated with an increased prevalence of NCDs among women. Other studies have also shown that consuming smokeless tobacco which is also a risk factor for oral cancers is a major problem associated with morbidity in Indian women particularly those with a lower socioeconomic status [37, 38]. The combined exposure of alcohol consumption and tobacco use was strongly associated with a higher prevalence of NCDs among women. The higher population-attributable risk of smoking, using smokeless tobacco and drinking alcohol for NCDs among women in the current study were noticeable and support the previous findings from India and other developing countries on the higher risk of smoking and alcohol consumption on hypertension and other NCDs [20, 39–41]. Thus, the results suggest a need for developing an efficient preventive strategy against the growing trend of NCDs through control of tobacco use and alcohol consumption. For example, as evidence suggests, a 10% increase in the price of tobacco reduces smoking by about 8% in low-and middle-income countries [42]. Similarly, an increase in taxation can be a potential strategy to control tobacco use especially among the poorest segment of the population.

Urbanization and adoption of unhealthy lifestyles that contribute to inappropriate food choices such as increased intake of sugar and fat led to an increase in body weight in the general population and women in particular [43]. Similarly, studies have shown maternal obesity as a major risk factor for gestational diabetes and pregnancy-induced hypertension in women [44, 45]. Consistently, the current analysis shows that women who were overweight or obese were more likely to have NCDs. This association of indices of obesity with an increased risk for NCDs among women of reproductive age confirms the results of other studies in both developed and developing countries [12, 46, 47]. Further, findings of overweight and obesity as factors associated with NCDs agree with the general view that body fat in humans is linked to a higher rate of cardiovascular diseases [48, 49].

In addition, lower levels of education, increasing age, being divorced /separated /widowed were associated with a higher risk of NCDs among women of reproductive age. More so, the association of increasing age with the higher risk of NCDs can be explained by the negative biological effects as women grow older [50, 51]. Furthermore, the associations of education and marital status with NCDs were similar to past studies showing that lower levels of education and being divorced, separated or widowed increased the odds of having NCDs in comparison to uneducated and never married women [52, 53]. Factors such as hormonal changes in reproductive cycle, chronic stress, women’s sociocultural vulnerability and marital relationship satisfaction might have influenced the observed association of marital status with NCD prevalence [54, 55]. Future research is necessary to confirm this association and explore the underlying mechanisms. On the other hand, the current study depicts that the chances of having NCDs were higher among women with higher household economic status which was also observed in previous studies [3, 13, 56]. This however, could be attributed to the lower levels of healthcare utilisation and less likelihood of women from poor socioeconomic background to be diagnosed and report medical conditions.

The calculation of population-attributable risks of smoking, consuming smokeless tobacco and alcohol consumption was the major strength of the current study. Also, this study used nationally representative secondary data and the findings are generalizable for the women of reproductive age in India. However, a major drawback is that the cross-sectional study design used cannot adequately establish causality. Moreover, the self-reported nature of data on NCDs not diagnosed or tested is subject to several biases which have influenced the current findings. The lack of information on several diseases and many behavioural factors limited this study to reveal the evidence around NCDs’ risk factors with sufficient depth. Future work might include the longitudinal assessment of NCDs with more diseases and their combinations along with assessing the population-attributable risks of several behavioural factors for increased NCD prevalence and for particular diseases in women.

## Conclusion

The findings revealed that smoking and using smokeless tobacco and alcohol consumption were risk factors of NCDs in women. The study findings urge health decision-makers to invest in women’s health especially those who are more exposed to the risk factors of having NCDs. The findings also alarm the focus of maternal and child health programs on NCDs’ risk factors like maternal obesity, due to their adverse health consequences on their children too. Also, the coexistence of higher levels of tobacco use and alcohol consumption requires different strategies to address the vulnerability of women towards NCDs. The screening and early detection of several NCDs such as diabetes, hypertension and heart disease should strongly be emphasised especially among those who smoke or chew tobacco and consume alcohol. Furthermore, interventions that focus on modifiable factors such as smoking and alcohol consumption, and related obesity can help prevent the increasing burden of NCDs among women in India.

## Supplementary Information

**Additional file 1: Table S1.** Logistic regression estimates for Diabetes, Asthama, Heart diseases, Cancer, Hypertension by background characteristics among women aged 15-49 years in India, 2015-16. **Table S2.** Population attributable risk for Diabetes among women aged 15-49 years in India, 2015-16. **Table S3.** Population attributable risk for Asthama among women aged 15-49 years in India, 2015-16. **Table S4.** Population attributable risk for Thyroid among women aged 15-49 years in India, 2015-16. **Table S5.** Population attributable risk for Heart diseases among women aged 15-49 years in India, 2015-16. **Table S6.** Population attributable risk for Cancer among women aged 15-49 years in India, 2015-16. **Table S7.** Population attributable risk for hypertension among women aged 15-49 years in India, 2015-16.

## Data Availability

The study utilizes secondary source of data which is freely available in public domain through dhsprogram.com.

## Abbreviations

NCDs: Non-Communicable Diseases
NFHS: National Family Health Survey
MoHFW: Ministry of Health and Family Welfare
IIPS: International Institute for Population Sciences
PSUs: Primary Sampling Units
CEBs: Census Enumeration Blocks
CAPI: Computer Assisted Personal Interviewing
BMI: Body mass index
PAR: Population Attributable Risk
AOR: Adjusted Odds Ratio
RR: Relative Risk
CI: Confidence Interval
VIF: Variance inflation factor

## References

[1] WHO, World Health Organization (2013). Global action plan for the prevention and control of noncommunicable diseases 2013-2020.

[2] Ezzati M, Riboli E (2013). Behavioral and dietary risk factors for noncommunicable diseases. N Engl J Med.

[3] Bista B, Dhungana RR, Chalise B (2020). Prevalence and determinants of noncommunicable diseases risk factors among reproductive aged women of Nepal: results from Nepal demographic health survey 2016. PLoS One.

[4] Khuwaja AK, Kadir MM (2010). Gender differences and clustering pattern of behavioural risk factors for chronic non-communicable diseases: community-based study from a developing country. Chronic Illn.

[5] Godfrey KM, Gluckman PD, Hanson MA (2010). Developmental origins of metabolic disease: life course and intergenerational perspectives. Trends Endocrinol Metab.

[6] Ronksley PE, Brien SE, Turner BJ (2011). Association of alcohol consumption with selected cardiovascular disease outcomes: a systematic review and meta-analysis. BMJ.

[7] Holmes MV, Dale CE, Zuccolo L (2014). Association between alcohol and cardiovascular disease: Mendelian randomisation analysis based on individual participant data. BMJ.

[8] Djoussé L, Faha D, Lee I (2016). Alcohol consumption and risk of cardiovascular disease and mortality in Women: potential mediating mechanisms. Circ Am Heart Assoc.

[9] Puddey IB, Rakic V, Dimmitt SB (1999). Influence of pattern of drinking on cardiovascular disease and cardiovascular risk factors - a review. Addiction.

[10] Allen LN, Smith RW, Simmons-Jones F (2020). Addressing social determinants of noncommunicable diseases in primary care: a systematic review. Bull World Health Organ.

[11] Ng SW, Zaghloul S, Ali HI (2011). The prevalence and trends of overweight, obesity and nutrition-related non-communicable diseases in the Arabian Gulf States. Obes Rev.

[12] Bernal RTI, Felisbino-Mendes MS, de Carvalho QH, et al. Indicators of chronic noncommunicable diseases in women of reproductive age that are beneficiaries and non-beneficiaries of Bolsa Família. Rev Bras Epidemiol. 2019;22. 10.1590/1980-549720190012.supl.2. 10.1590/1980-549720190012.supl.1 PMC6892639 31596383

[13] Yaya S, Uthman OA, Ekholuenetale M (2018). Socioeconomic inequalities in the risk factors of noncommunicable diseases among women of reproductive age in sub-Saharan Africa: a multi-country analysis of survey data. Front Public Health.

[14] World Health Organisation (2002). Women and the Rapid Rise of Noncommunicable Diseases.

[15] Bombard JM, Dietz PM, Galavotti C (2012). Chronic diseases and related risk factors among low-income mothers. Matern Child Health J.

[16] Bhagyalaxmi A, Atul T, Shikha J (2013). Prevalence of risk factors of non-communicable diseases in a district of Gujarat, India. J Health Popul Nutr.

[17] Chopra SM, Misra A, Gulati S (2013). Overweight , obesity and related non-communicable diseases in Asian Indian girls and women. Eur J Clin Nutr.

[18] Upadhyay RP (2012). An overview of the burden of non-communicable diseases in India. Iran J Public Health.

[19] International Institute for Population Sciences (IIPS) and ICF (2017). National Family Health Survey (NFHS-4).

[20] Anchala R, Kannuri NK, Pant H (2014). Hypertension in India: a systematic review and meta-analysis of prevalence, awareness, and control of hypertension. J Hypertens.

[21] McKenna SP. Measuring patient-reported outcomes: moving beyond misplaced common sense to hard science. BMC Med. 2011. 10.1186/1741-7015-9-86. 10.1186/1741-7015-9-86 PMC3170214 21756344

[22] World Health Organization. Obesity and overweight: Fact sheet. WHO Media Centre. 2020.

[23] Srivastava S, Kumar S (2021). Does socio-economic inequality exist in micro-nutrients supplementation among children aged 6–59 months in India? Evidence from National Family Health.

[24] Srivastava S, Singh SK, Kumar M (2021). Distinguishing between household headship with and without power and its association with subjective well-being among older adults: an analytical cross-sectional study in India. BMC Geriatr.

[25] Srivastava S, Debnath P, Shri N (2021). The association of widowhood and living alone with depression among older adults in India. Sci Rep.

[26] Osborne J, King JE. Binary logistic regression. In: Best Practices in Quantitative Methods. In: SAGE Publications, Inc. New York; 2011. p. 358–84.

[27] Lewis-Beck M, Bryman A, Liao TF (2004). Variance inflation factors. The SAGE Encyclopedia of Social Science Research Methods.

[28] Muhammad T, Govindu M, Srivastava S (2021). Relationship between chewing tobacco, smoking, consuming alcohol and cognitive impairment among older adults in India: a cross-sectional study. BMC Geriatr.

[29] Srivastava S, Muhammad T. Violence and associated health outcomes among older adults in India: a gendered perspective. SSM Popul Health. 2020;12. 10.1016/j.ssmph.2020.100702. 10.1016/j.ssmph.2020.100702 PMC7708932 33304986

[30] Chauhan S, Srivastava S, Kumar P, et al. Interaction of substance use with physical activity and its effect on depressive symptoms among adolescents. J Subst Abus. 2021;26(5):524–30.

[31] Van Der Weele TJ, Knol MJ (2014). A tutorial on interaction. Epidemiol Methods.

[32] Srivastava S, Shankar Mishra P, Sinha D, et al. Population attributable risk for breastfeeding practices on diarrhea and acute respiratory infections among children aged 0–23 months in India – what we know and we do not know? Child Youth Serv Rev. 2020. 10.1016/j.childyouth.2020.105531.

[33] Newson R. REGPAR: Stata module to compute population attributable risks from binary regression models.

[34] Anand K, Shah B, Yadav K (2007). Are the urban poor vulnerable to non-communicable diseases? A survey of risk factors for non-communicable diseases in urban slums of Faridabad. Natl Med J India.

[35] Kinra S, Bowen LJ, Lyngdoh T, et al. Socio-demographic patterning of non-communicable disease risk factors in rural India: a cross sectional study. BMJ. 2010. 10.1136/bmj.c4974. 10.1136/bmj.c4974 PMC2946988 20876148

[36] Nethan S, Sinha D, Mehrotra R (2017). Non communicable disease risk factors and their trends in India. Asian Pac J Cancer Prev.

[37] Joseph I, Rooban T, Ranganathan K (2017). Tobacco use, Oral Cancer screening, and Oral disease burden in Indian women. Indian J Dent Res.

[38] Nair S, Schensul JJ, Begum S (2015). Use of smokeless tobacco by Indian women aged 18-40 years during pregnancy and reproductive years. PLoS One.

[39] Boutayeb A, Boutayeb S (2005). The burden of non communicable diseases in developing countries. Int J Equity Health.

[40] Prabhakaran D, Jeemon P, Sharma M (2018). The changing patterns of cardiovascular diseases and their risk factors in the states of India: the global burden of disease study 1990–2016. Lancet Glob Health.

[41] Mpofu JJ, de Moura L, Farr SL (2016). Associations between noncommunicable disease risk factors, race, education, and health insurance status among women of reproductive age in Brazil - 2011. Prev Med Rep.

[42] Jha P, Chaloupka FJ (2000). Tobacco control in developing countries.

[43] Yaya S, Ekholuenetale M, Bishwajit G (2018). Differentials in prevalence and correlates of metabolic risk factors of non- communicable diseases among women in sub-Saharan Africa : evidence from 33 countries. BMC Public Health.

[44] Athukorala C, Rumbold AR, Willson KJ, et al. The risk of adverse pregnancy outcomes in women who are overweight or obese. BMC Pregnancy Childbirth. 2010;10. 10.1186/1471-2393-10-56. 10.1186/1471-2393-10-56 PMC2949787 20849609

[45] Ayensu J, Annan RA, Edusei A (2016). Impact of maternal weight on pregnancy outcomes: a systematic review. Nutr Food Sci.

[46] Werneck AO, Oyeyemi AL, Szwarcwald CL, et al. Body mass index trajectories and noncommunicable diseases in women: the role of leisure time physical activity. Am J Hum Biol. 2021;33(3):e23492. 10.1002/ajhb.23492 32885890

[47] Kruger HS, Venter CS, Vorster HH (2001). Obesity in African women in the north West Province, South Africa is associated with an increased risk of non-communicable diseases: the THUSA study. Br J Nutr.

[48] Gnatiuc L, Alegre-Díaz J, Halsey J (2017). Adiposity and blood pressure in 110 000 Mexican adults. Hypertension.

[49] Song X, Jousilahti P, Stehouwer CDA (2013). Comparison of various surrogate obesity indicators as predictors of cardiovascular mortality in four European populations. Eur J Clin Nutr.

[50] Kuri-Morales P, Emberson J, Alegre-Díaz J (2009). The prevalence of chronic diseases and major disease risk factors at different ages among 150 000 men and women living in Mexico City: cross-sectional analyses of a prospective study. BMC Public Health.

[51] Oliver JE, Silman AJ (2009). Why are women predisposed to autoimmune rheumatic diseases?. Arthritis Res Ther.

[52] Hosseinpoor AR, Bergen N, Mendis S (2012). Socioeconomic inequality in the prevalence of noncommunicable diseases in low- and middle-income countries: results from the world health survey. BMC Public Health.

[53] Yepes M, Maurer J, Viswanathan B, et al. Potential reach of mhealth versus traditional mass media for prevention of chronic diseases: Evidence from a nationally representative survey in a middle-income country in Africa. J Med Internet Res. 2016;18. 10.2196/jmir.5592. 10.2196/jmir.5592 PMC4893150 27207074

[54] Segawa HK, Uematsu H, Dorji N (2021). Gender with marital status, cultural differences, and vulnerability to hypertension: findings from the national survey for noncommunicable disease risk factors and mental health using WHO STEPS in Bhutan. PLoS One.

[55] Wekesah FM, Nyanjau L, Kibachio J, et al. Individual and household level factors associated with presence of multiple non-communicable disease risk factors in Kenyan adults. BMC Public Health. 2018;18. 10.1186/s12889-018-6055-8. 10.1186/s12889-018-6055-8 PMC6219015 30400905

[56] Dalal S, Beunza JJ, Volmink J (2011). Non-communicable diseases in sub-Saharan Africa: what we know now. Int J Epidemiol.

