# Supplementary material for: Relationship between tobacco use, alcohol consumption and non-communicable diseases among women in India: evidence from National Family Health Survey-2015-16
Source: BMC Public Health. 2022 Apr 11;22:713. doi: 10.1186/s12889-022-13191-z (PMC8996590; doi:10.1186/s12889-022-13191-z)
Supplement: Supplementary file 1 — Additional file 1: Table S1. Logistic regression estimates for Diabetes, Asthama, Heart diseases, Cancer, Hypertension by background characteristics among women aged 15-49 years in India, 2015-16. Table S2. Population attributable risk for Diabetes among women aged 15-49 years in India, 2015-16. Table S3. Population attributable risk for Asthama among women aged 15-49 years in India, 2015-16. Table S4. Population attributable risk for Thyroid among women aged 15-49 years in India, 2015-16. Table S5. Population attributable risk for Heart diseases among women aged 15-49 years in India, 2015-16. Table S6. Population attributable risk for Cancer among women aged 15-49 years in India, 2015-16. Table S7. Population attributable risk for hypertension among women aged 15-49 years in India, 2015-16. [file 12889_2022_13191_MOESM1_ESM.docx]

| **Table-S1.** Logistic regression estimates for Diabetes, Asthama, Heart diseases, Cancer, Hypertension by background characteristics among women aged 15-49 years in India, 2015-16 | | | | | |
| --- | --- | --- | --- | --- | --- |
| **Behavioural characteristics** | **Diabetes** | **Asthma** | **Heart diseases** | **Cancer** | **Hypertension** |
|  | **AOR (95% CI)** | **AOR (95% CI)** | **AOR (95% CI)** | **AOR (95% CI)** | **AOR (95% CI)** |
| **Smoke tobacco** |  |  |  |  |  |
| No | Ref. | Ref. | Ref. | Ref. | Ref. |
| Yes | 1.18*(1.09,1.28) | 1.82*(1.61,2.06) | 2.28*(2.06,2.52) | 1.89*(1.25,2.86) | 0.82*(0.77,0.88) |
| **Consume smokeless tobacco** |  |  |  |  |  |
| No | Ref. | Ref. | Ref. | Ref. | Ref. |
| Yes | 1.14*(1.1,1.18) | 1.38*(1.3,1.47) | 1.24*(1.17,1.32) | 0.81(0.64,1.03) | 0.97*(0.94,1) |
| **Alcohol consumption** |  |  |  |  |  |
| No | Ref. | Ref. | Ref. | Ref. | Ref. |
| Yes | 0.94*(0.88,1) | 1.28*(1.15,1.42) | 1.1(0.98,1.23) | 1.33(0.92,1.92) | 1.29*(1.23,1.35) |
| **Individual characteristics** |  |  |  |  |  |
| **Age (in years)** |  |  |  |  |  |
| 15-24 | Ref. | Ref. | Ref. | Ref. | Ref. |
| 25-34 | 1.65*(1.58,1.72) | 1.6*(1.49,1.71) | 1.83*(1.7,1.98) | 1.2(0.96,1.49) | 1.97*(1.91,2.04) |
| 35-49 | 3.53*(3.39,3.68) | 2.7*(2.52,2.9) | 3.48*(3.22,3.75) | 2.15*(1.73,2.67) | 4.67*(4.51,4.83) |
| **Educational status** |  |  |  |  |  |
| Not educated | Ref. | Ref. | Ref. | Ref. | Ref. |
| Primary | 1.03(1,1.07) | 1.08*(1.02,1.15) | 1.09*(1.02,1.15) | 1.01(0.81,1.25) | 0.99(0.97,1.02) |
| Secondary | 0.93*(0.91,0.96) | 0.93*(0.88,0.98) | 0.98(0.93,1.04) | 1.08(0.9,1.3) | 0.86*(0.84,0.88) |
| Higher | 0.83*(0.79,0.86) | 0.73*(0.67,0.79) | 0.75*(0.69,0.83) | 0.89(0.66,1.19) | 0.71*(0.68,0.74) |
| **Marital status** |  |  |  |  |  |
| Never married | Ref. | Ref. | Ref. | Ref. | Ref. |
| Currently married | 0.94*(0.9,0.98) | 0.88*(0.82,0.95) | 1(0.93,1.09) | 0.91(0.73,1.13) | 1.04*(1.01,1.08) |
| Others | 1.07*(1.01,1.14) | 1.03(0.93,1.14) | 1.18*(1.06,1.31) | 1.07(0.76,1.51) | 1.19*(1.13,1.25) |
| **Media exposure** |  |  |  |  |  |
| Not exposed | Ref. | Ref. | Ref. | Ref. | Ref. |
| Exposed | 1.04*(1.01,1.08) | 1.21*(1.14,1.28) | 1.24*(1.17,1.32) | 1.21(0.99,1.46) | 0.97*(0.95,1) |
| **Body Mass Index** |  |  |  |  |  |
| Underweight | Ref. | Ref. | Ref. | Ref. | Ref. |
| Normal | 1.08*(1.04,1.11) | 1.01(0.96,1.07) | 1.03(0.97,1.09) | 1.04(0.87,1.23) | 1.36*(1.32,1.4) |
| Overweight | 1.95*(1.88,2.03) | 1.4*(1.31,1.49) | 1.3*(1.21,1.39) | 1.02(0.8,1.28) | 2.75*(2.67,2.84) |
| Obese | 3.18*(3.04,3.32) | 1.83*(1.68,1.99) | 1.69*(1.54,1.85) | 1.06(0.75,1.48) | 4.11*(3.95,4.27) |
| **Household characteristic’s** |  |  |  |  |  |
| **Wealth status** |  |  |  |  |  |
| Poorest | Ref. | Ref. | Ref. | Ref. | Ref. |
| Poorer | 1.08*(1.04,1.12) | 1.08*(1.01,1.15) | 1.13*(1.06,1.21) | 0.9(0.73,1.11) | 1(0.97,1.03) |
| Middle | 1.16*(1.12,1.21) | 1(0.93,1.08) | 1.15*(1.07,1.23) | 0.87(0.69,1.1) | 1(0.97,1.03) |
| Richer | 1.3*(1.24,1.36) | 1.04(0.96,1.12) | 1.13*(1.05,1.23) | 1.01(0.78,1.3) | 1.06*(1.02,1.09) |
| Richest | 1.35*(1.29,1.42) | 1.06(0.97,1.16) | 0.97(0.88,1.06) | 1.05(0.78,1.4) | 1.02(0.98,1.06) |
| **Religion** |  |  |  |  |  |
| Hindu | Ref. | Ref. | Ref. | Ref. | Ref. |
| Muslim | 1.04*(1.01,1.07) | 0.91*(0.86,0.97) | 1.75*(1.66,1.84) | 1.21*(1,1.47) | 1.15*(1.12,1.18) |
| Christian | 1.06*(1.01,1.11) | 1.24*(1.14,1.35) | 1.7*(1.56,1.86) | 1.22(0.91,1.63) | 0.82*(0.79,0.86) |
| Others | 0.96(0.91,1.01) | 0.99(0.9,1.09) | 0.99(0.89,1.09) | 0.78(0.54,1.12) | 1.09*(1.05,1.13) |
| **Caste** |  |  |  |  |  |
| Scheduled Caste | Ref. | Ref. | Ref. | Ref. | Ref. |
| Scheduled Tribe | 1(0.96,1.04) | 0.9*(0.84,0.97) | 0.8*(0.74,0.87) | 0.94(0.74,1.2) | 1.08*(1.05,1.12) |
| Other Backward Class | 0.96*(0.93,0.99) | 0.99(0.94,1.05) | 0.85*(0.8,0.9) | 0.88(0.73,1.06) | 0.96*(0.93,0.98) |
| Others | 1.01(0.97,1.04) | 1.12*(1.05,1.19) | 1.09*(1.02,1.16) | 1.11(0.9,1.36) | 1.03*(1,1.06) |
| **Place of residence** |  |  |  |  |  |
| Urban | Ref. | Ref. | Ref. | Ref. | Ref. |
| Rural | 1(0.97,1.02) | 1.07*(1.02,1.12) | 1.13*(1.08,1.19) | 1.28*(1.08,1.51) | 1.04*(1.01,1.06) |
| **Regions** |  |  |  |  |  |
| North | Ref. | Ref. | Ref. | Ref. | Ref. |
| Central | 1.23*(1.19,1.28) | 1.31*(1.22,1.4) | 1.13*(1.06,1.2) | 1.07(0.85,1.35) | 0.95*(0.92,0.97) |
| East | 1.41*(1.36,1.46) | 1.78*(1.66,1.91) | 1.14*(1.07,1.22) | 2.46*(1.97,3.07) | 0.89*(0.87,0.92) |
| North East | 1.31*(1.25,1.37) | 1.16*(1.07,1.27) | 1.2*(1.11,1.3) | 1.29(0.97,1.72) | 1.57*(1.52,1.62) |
| West | 1.09*(1.04,1.14) | 1.51*(1.39,1.64) | 0.4*(0.36,0.45) | 1.03(0.75,1.41) | 0.98(0.94,1.01) |
| South | 1.35*(1.3,1.4) | 2.33*(2.18,2.49) | 0.78*(0.72,0.84) | 2.03*(1.61,2.57) | 0.84*(0.81,0.87) |
| Ref: Reference; AOR: Adjusted odds ratio; CI: Confidence interval; *if p<0.05 | | | | | |

| **Table S2**: Population attributable risk for Diabetes among women aged 15-49 years in India, 2015-16 | |
| --- | --- |
| *Population attributable risk (PAR)* | |
| **Behavioural factors** | **Diabetes** |
| **Smoke tobacco** |  |
| No | 0.014*(0.013,0.014) |
| Yes | 0.020*(0.018,0.024) |
| PAR | 0.006*(0.001,0.005) |
| **Chew Tobacco** |  |
| No | 0.014*(0.013,0.014) |
| Yes | 0.015*(0.014,0.016) |
| PAR | 0.001(0.001,0.002) |
| **Alcohol consumption** |  |
| No | 0.014*(0.013,0.014) |
| Yes | 0.016*(0.014,0.019) |
| PAR | 0.003*(0.001,0.005) |
| *CI: Confidence Interval; The analysis was controlled for individual and household characteristics* | |

| **Table S3**: Population attributable risk for Asthama among women aged 15-49 years in India, 2015-16 | |
| --- | --- |
| *Population attributable risk (PAR)* | |
| **Behavioural factors** | **Asthama** |
| **Smoke tobacco** |  |
| No | 0.016*(0.016,0.016) |
| Yes | 0.028*(0.025,0.032) |
| PAR | 0.012*(0.009,0.012) |
| **Chew Tobacco** |  |
| No | 0.016*(0.016,0.016) |
| Yes | 0.021*(0.02,0.022) |
| PAR | 0.005*(0.004,0.006) |
| **Alcohol consumption** |  |
| No | 0.016*(0.016,0.016) |
| Yes | 0.020*(0.018,0.022) |
| PAR | 0.004*(0.002,0.004) |
| *CI: Confidence Interval; The analysis was controlled for individual and household characteristics* | |

| **Table S4**: Population attributable risk for Thyroid among women aged 15-49 years in India, 2015-16 | |
| --- | --- |
| *Population attributable risk (PAR)* | |
| **Behavioural factors** | **Goitre or any other thyroid disorder** |
| **Smoke tobacco** |  |
| No | 0.019*(0.019,0.019) |
| Yes | 0.027*(0.023,0.031) |
| PAR | 0.008*(0.004,0.011) |
| **Chew Tobacco** |  |
| No | 0.019*(0.019,0.019) |
| Yes | 0.021*(0.019,0.022) |
| PAR | 0.002*(0.001,0.003) |
| **Alcohol consumption** |  |
| No | 0.019*(0.019,0.019) |
| Yes | 0.018*(0.016,0.021) |
| PAR | 0.001(0.001,0.003) |
| *CI: Confidence Interval; The analysis was controlled for individual and household characteristics* | |

| **Table S5**: Population attributable risk for Heart diseases among women aged 15-49 years in India, 2015-16 | |
| --- | --- |
| *Population attributable risk (PAR)* | |
| **Behavioural factors** | **Heart diseases** |
| **Smoke tobacco** |  |
| No | 0.015*(0.015,0.015) |
| Yes | 0.032*(0.029,0.035) |
| PAR | 0.017*(0.014,0.020) |
| **Chew Tobacco** |  |
| No | 0.015*(0.015,0.015) |
| Yes | 0.018*(0.017,0.019) |
| PAR | 0.003*(0.002,0.004) |
| **Alcohol consumption** |  |
| No | 0.015*(0.015,0.015) |
| Yes | 0.016*(0.015,0.018) |
| PAR | 0.001(0.001,0.003) |
| *CI: Confidence Interval; The analysis was controlled for individual and household characteristics* | |

| **Table S6**: Population attributable risk for Cancer among women aged 15-49 years in India, 2015-16 | |
| --- | --- |
| *Population attributable risk (PAR)* | |
| **Behavioural factors** | **Cancer** |
| **Smoke tobacco** |  |
| No | 0.001*(0.001,0.001) |
| Yes | 0.003*(0.002,0.004) |
| PAR | 0.001(0.001,0.002) |
| **Chew Tobacco** |  |
| No | 0.001*(0.001,0.001) |
| Yes | 0.001*(0.001,0.001) |
| PAR | 0.001(0.001,0.005) |
| **Alcohol consumption** |  |
| No | 0.001*(0.001,0.001) |
| Yes | 0.002*(0.001,0.003) |
| PAR | 0.001(0.001,0.002) |
| *CI: Confidence Interval; The analysis was controlled for individual and household characteristics* | |

| **Table S7**: Population attributable risk for hypertension among women aged 15-49 years in India, 2015-16 | |
| --- | --- |
| *Population attributable risk (PAR)* | |
| **Behavioural factors** | **Hypertension** |
| **Smoke tobacco** |  |
| No | 0.091*(0.09,0.091) |
| Yes | 0.077*(0.072,0.082) |
| PAR | -0.014*-(0.018,-009) |
| **Chew Tobacco** |  |
| No | 0.091*(0.09,0.091) |
| Yes | 0.089*(0.087,0.091) |
| PAR | -0.002*(-0.004,0.001) |
| **Alcohol consumption** |  |
| No | 0.091*(0.09,0.091) |
| Yes | 0.111*(0.107,0.115) |
| PAR | 0.020*(0.016,0.025) |
| *CI: Confidence Interval; The analysis was controlled for individual and household characteristics* | |
